# Mosaic *KRAS* variants in childhood-onset systemic lupus erythematosus

**DOI:** 10.70962/jhi.20260098

**Published:** 2026-10-23

**Authors:** Hitoshi Irabu, Madoka Nishimura, Fumiaki Sakura, Takaki Asano, Maho Hatano, Futaba Miyaoka, Shuya Kaneko, Asami Shimbo, Yuko Akutsu, Ryosuke Wakatsuki, Dan Tomomasa, Sanami Takada, Yuko Hayashi, Satoshi Okada, Masaaki Mori, Tomohiro Morio, Masatoshi Takagi, Hirokazu Kanegane, Masaki Shimizu

**Affiliations:** 1 Department of Pediatrics and Developmental Biology, Graduate School of Medical and Dental Sciences, Institute of Science Tokyo, Tokyo, Japan; 2 Department of Pediatrics, Graduate School of Medical Sciences, Kumamoto University, Kumamoto, Japan; 3 Department of Pediatrics, Hiroshima University Graduate School of Biomedical & Health Sciences, Hiroshima, Japan; 4 Laboratory of Immunology and Molecular Medicine, Advanced Research Initiative, Institute of Science Tokyo, Tokyo, Japan; 5 Department of Pediatrics, Neonatal and Maternal Medicine, Graduate School of Medical and Dental Sciences, Institute of Science Tokyo, Tokyo, Japan; 6 Department of Lifelong Immunotherapy, Institute of Biomedical Engineering, Institute of Science Tokyo, Tokyo, Japan; 7 Department of Child Health and Development, Graduate School of Medical and Dental Sciences, Institute of Science Tokyo, Tokyo, Japan

## Abstract

Clinical targeted sequencing identified low-VAF *KRAS* variants in two of 26 children with childhood-onset systemic lupus erythematosus (SLE). Both had severe inflammatory manifestations but lacked Noonan syndrome features, and neither developed persistent lymphoproliferation suggestive of RAS-associated autoimmune lymphoproliferative disease during follow-up. These findings suggest that *KRAS* mosaicism may contribute to the development or severity of childhood-onset SLE.

## Teaser

This study identifies low-VAF *KRAS *variants in two children with systemic lupus erythematosus and confirms both variants in independent blood samples. The findings suggest that RAS pathway mosaicism may contribute to childhood lupus beyond classic RAS-associated autoimmune lymphoproliferative disease.

## Introduction

Systemic lupus erythematosus (SLE) reflects interactions between genetic and environmental factors. Genetic contributions are stronger in childhood-onset SLE (cSLE), and a subset results from germline variants causing inborn errors of immunity or monogenic lupus (1).

Like germline variants, mosaic variants can disrupt immune regulation. For example, activating mosaic *KRAS* or *NRAS* variants are known to cause RAS-associated autoimmune lymphoproliferative disease (RALD), characterized by cytopenias, lymphoproliferation, and autoimmunity (2). However, relatively few mosaic variants associated with immune dysregulation have been described.

In this study, we analyzed inborn error of immunity–associated genes by next-generation sequencing in whole-blood samples from 26 Japanese children with cSLE and identified low variant allele frequency (VAF) *KRAS* variants suggestive of mosaicism in two patients. We subsequently assessed these variants in blood, hair, saliva, urine, and nail samples by amplicon sequencing, detecting low-level alternate reads in several nonblood specimens. We then examined the potential clinical significance of *KRAS* mosaicism in SLE.

## Results

Among 26 Japanese children with cSLE evaluated for inborn errors of immunity, initial clinical targeted sequencing identified low-VAF *KRAS* variants in two patients. Table 1 summarizes the clinical characteristics of the whole cohort and, separately, of the two *KRAS*-positive patients and the remaining 24 patients. Both had serositis, lupus nephritis, leukopenia, hypocomplementemia, and SLE Disease Activity Index 2000 (SLEDAI-2K) scores of 15 and 14. Neither patient had dysmorphic, cardiac, or growth features of Noonan syndrome or another RASopathy.

**Table 1.** Clinical characteristics of the study cohort according to detection of *KRAS* mosaicism

Characteristic	All patients (*n* = 26)	*KRAS* mosaicism detected (*n* = 2)	No *KRAS* mosaicism detected (*n* = 24)
Female sex, *n* (%)	23 (88.5)	2 (100)	21 (87.5)
Age at onset, years, median (range)	11 (1–16)	9 (7–11)	11 (1–16)
Onset before 6 years, *n* (%)	4 (15.4)	0 (0.0)	4 (16.7)
Serositis, *n* (%)	3 (11.5)	2 (100)	1 (4.2)
Lupus nephritis, *n* (%)	19 (73.1)	2 (100)	17 (70.8)
Neurologic involvement, *n* (%)	6 (23.1)	0 (0.0)	6 (25.0)
Macrophage activation syndrome, *n* (%)	1 (3.8)	1 (50)	0 (0.0)
Hemolytic anemia, *n* (%)	4 (15.4)	0 (0.0)	4 (16.7)
Leukopenia, *n* (%)	15 (57.7)	2 (100)	13 (54.2)
Thrombocytopenia, *n* (%)	4 (15.4)	0 (0.0)	4 (16.7)
Hypocomplementemia, *n* (%)	18 (69.2)	2 (100)	16 (66.7)
SLEDAI-2K score, median (range)	12.5 (5–20)	14.5 (14–15)	11.5 (5–20)
Family history of systemic autoimmune disease, *n* (%)^a^	6 (23.1)	0 (0.0)	6 (25.0)
Alive at last follow-up, *n* (%)	26 (100.0)	2 (100)	24 (100.0)

a Family history denotes a recorded relative with SLE or rheumatoid arthritis.

Patient 1 was a 7-year-old girl who presented with fever, dyspnea, lymphadenopathy, and severe chest pain requiring hospitalization. Echocardiography revealed pericardial effusion, chest computed tomography showed bilateral interstitial pneumonia, and serositis was refractory to steroid pulse therapy. Clinical sequencing detected *KRAS*NM_004985.5:c.64C>A, p.Gln22Lys, classified as likely pathogenic in ClinVar, in blood at 10.5% VAF (depth, 258). Amplicon sequencing of an independent blood sample obtained ∼9 mo later showed 9.57% VAF (4,499/47,007 reads); saliva, urine, nail, and hair obtained concurrently showed 0.947%, 0.296%, 0.537%, and 0.133%, respectively (Table 2). Intravenous cyclophosphamide induced remission, maintained with mycophenolate mofetil and hydroxychloroquine (HCQ). At ∼62 mo after onset, she remained in complete remission on HCQ monotherapy (200 mg/day), without persistent lymphadenopathy, splenomegaly, other lymphoproliferation, or malignancy.

**Table 2.** Amplicon-based deep sequencing of *KRAS* variants in multiple specimens

​	*KRAS c.64C>A (p.Gln22Lys)*		*KRAS c.40G>A (p.Val14Ile)*	
Sample	Patient 1	c.64 background in patient 2	Patient 2	c.40 background in patient 1
Blood	4,499/47,007 (9.57%)	38/46,975 (0.08%)	6,789/47,005 (14.44%)	94/47,021 (0.20%)
Saliva	437/46,126 (0.95%)	81/46,409 (0.18%)	4,113/46,412 (8.86%)	80/46,138 (0.17%)
Urine	138/46,669 (0.30%)	66/46,915 (0.14%)	799/46,920 (1.70%)	29/46,676 (0.06%)
Nail	254/47,318 (0.54%)	143/46,772 (0.31%)	441/46,804 (0.94%)	81/47,291 (0.17%)
Hair	61/45,838 (0.13%)	0/46,661 (0.00%)	237/46,654 (0.51%)	182/45,831 (0.40%)

Values are shown as alternate reads/(reference + alternate reads), with variant allele fractions in parentheses. For each variant position, background was assessed in the other patient, who did not carry that variant, using identical mapping- and base-quality filtering. Each specimen was analyzed once; no dedicated negative controls or technical replicates were included, and the analytical limit of detection was not established.

Patient 2 was an 11-year-old girl who developed fever, a chest rash, and pharyngitis. By day 7, she had pancytopenia, markedly elevated transaminases, hyperferritinemia (8,495 ng/ml), and bone marrow hemophagocytosis, consistent with macrophage activation syndrome; steroid pulse therapy was effective. Clinical sequencing detected *KRAS*NM_004985.5:c.40G>A, p.Val14Ile, classified as pathogenic in ClinVar, in blood at 17.5% VAF (depth, 348). Amplicon sequencing of an independent blood sample obtained ∼21 mo later showed 14.4% VAF (6,789/47,005 reads); saliva, urine, nail, and hair obtained concurrently showed 8.86%, 1.70%, 0.942%, and 0.508%, respectively (Table 2). At ∼45 mo after onset, she remained in remission on HCQ (100 and 200 mg on alternate days), mycophenolate mofetil (1,000 mg/day), and belimumab (200 mg every 2 wk), without persistent lymphadenopathy, splenomegaly, other lymphoproliferation, or malignancy.

**Figure 1.**
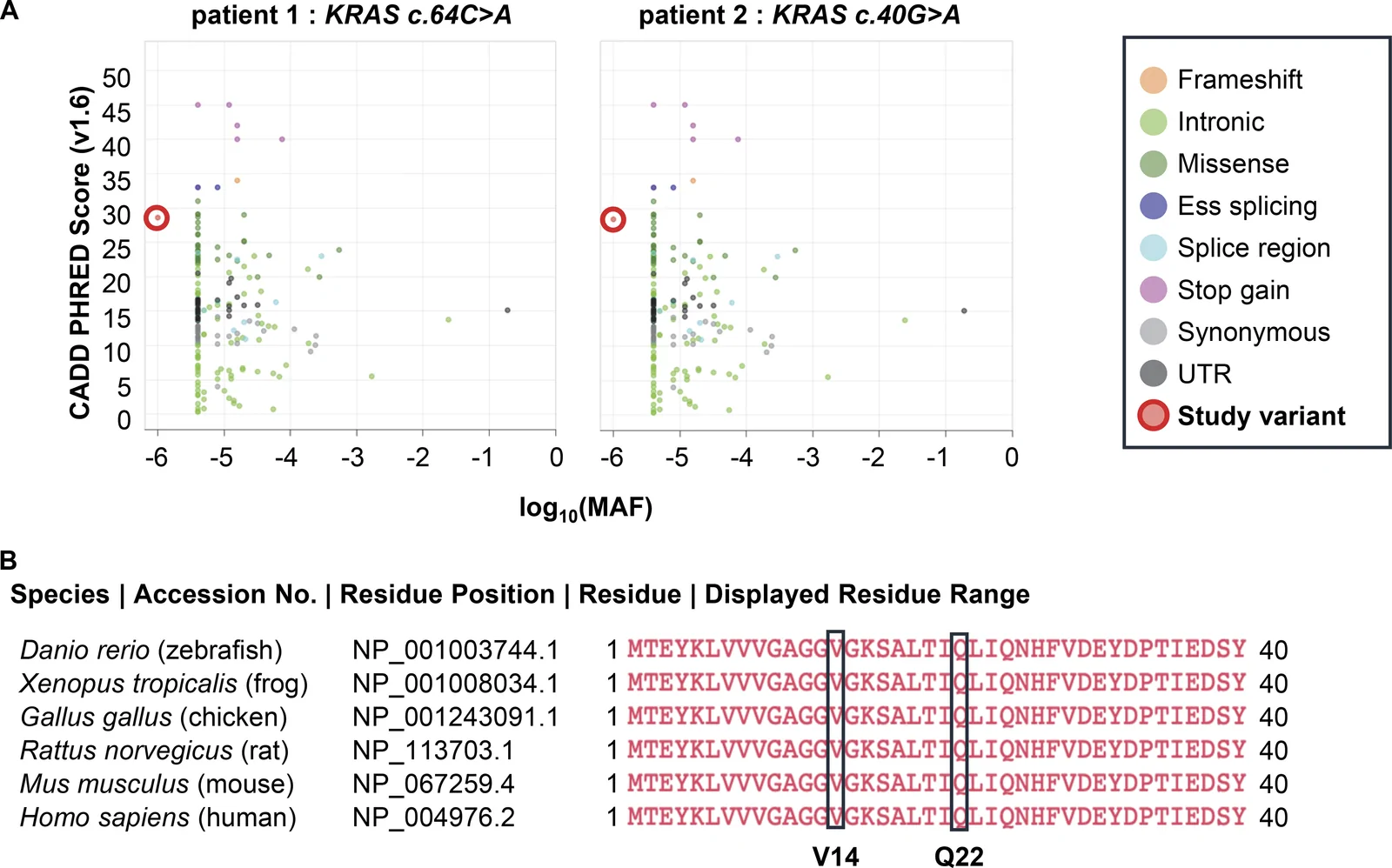
**In silico characterization and evolutionary conservation of *KRAS* variants. (A)** PopViz plots showing CADD scores and population allele frequencies of *KRAS* variants. The variants identified in patient 1 (NM_004985.5:c.64C>A, p.Gln22Lys) and patient 2 (NM_004985.5:c.40G>A, p.Val14Ile) are indicated by red open circles. Because neither variant had a reported allele frequency in the population dataset used for the PopViz analysis, both were plotted at log10(MAF) = −6 for visualization. Their genomic coordinates based on GRCh38, reference and alternate alleles in the genomic orientation, and CADD raw and PHRED scores are provided in Table 3. **(B)** Cross-species alignment of *KRAS* protein sequences. Val14 and Gln22, indicated by boxes, are conserved across the vertebrate species examined. MAF, minor allele frequency.

Both variants had Combined Annotation Dependent Depletion (CADD) v1.6 PHRED scores above 28 (Fig. 1 A and Table 3) and affected conserved residues (Fig. 1 B). At the corresponding nonvariant *KRAS* position in the other patient, specimen-matched empirical background VAFs after identical filtering ranged from 0% to 0.397% (Table 2). The repeat-blood VAFs in both patients and the saliva and urine VAFs in patient 2 were clearly above this background, supporting the presence of the variants in these specimens. Several other nonblood VAFs were only modestly above background, whereas values within the observed background range could not be confidently distinguished from sequencing noise. Because each specimen was analyzed only once, without replicates or an established limit of detection, repeat testing would be required to validate these low-level signals.

**Table 3.** Genomic coordinates and CADD scores of the identified *KRAS* variants

Patient	Variant	Chromosome	Position	Reference allele	Alternate allele	CADD raw score	CADD PHRED score
Patient 1	NM_004985.5:c.64C>A (p.Gln22Lys)	12	25245321	G	T	5.11247	28.6
Patient 2	NM_004985.5:c.40G>A (p.Val14Ile)	12	25245345	C	T	5.08367	28.4

Genomic coordinates are based on GRCh38. Reference and alternate alleles are shown in the genomic orientation. CADD scores were obtained using CADD v1.6.

The initial and follow-up blood samples were analyzed using different assays with substantially different sequencing depths; the two blood values were therefore not directly comparable, and longitudinal clonal dynamics could not be assessed. The variant findings did not influence treatment decisions, and formal genetic counseling had not been performed at the time of this report.

## Discussion

Independent amplicon sequencing confirmed low-VAF *KRAS* variants in separate blood samples from two children with cSLE. Somatic activating *KRAS* or *NRAS* variants have been reported in classic RALD and in lupus-like autoimmunity or SLE (2, 3). Neither patient developed persistent lymphoproliferation, suggesting that mosaic RAS pathway variants may also occur in cSLE without characteristic features of RALD. Mosaic variants in genes associated with immune dysregulation may therefore be underrecognized contributors to SLE and related immune-mediated disorders.

Both patients had severe inflammatory manifestations: refractory serositis with interstitial pneumonia in patient 1 and macrophage activation syndrome in patient 2. Severe or treatment-refractory disease has also been reported in SLE with somatic RAS variants (3). Both p.Val14Ile and p.Gln22Lys have been shown to increase RAS/MAPK signaling in a published cell-based assay (4); however, we did not perform functional analyses using patient-derived cells. The phenotypic divergence from classic RALD despite the reported activating effects of these variants may reflect differences in the developmental timing, tissue distribution, or lineage-specific burden of mosaicism. Because VAFs were not assessed in sorted immune-cell subsets, the distribution of the mutant clones among hematopoietic lineages remains unknown. With only two patients, causality and effects on cSLE severity cannot be established; these findings remain hypothesis generating.

Alternate reads were detected in blood and nonblood specimens, raising the possibility that mosaicism extends beyond the hematopoietic compartment. However, because several VAFs approached the empirical background and the cellular composition differed among specimens, no conclusions can be drawn regarding tissue distribution or lineage enrichment. Germ cells were not examined, and gonosomal mosaicism therefore cannot be excluded. The potential for transmission to offspring should be addressed in genetic counseling.

Neither variant passed the standard germline-calling filter; detection of low-VAF mosaic variants may therefore require high-depth sequencing and mosaic-aware variant calling. The prognostic value of *KRAS* mosaicism remains unknown. Larger cohorts, long-term follow-up, sorted-cell analyses, and patient-derived functional assays are needed to determine whether mosaic variants contribute to cSLE pathogenesis and to define the phenotypic spectrum of RAS pathway mosaicism.

These cases support a phenotypic spectrum of *KRAS* mosaicism extending beyond classic RALD and suggest a potential contribution to severe cSLE. Mosaic-aware analysis of sequencing data may be worth considering in children with severe or atypical cSLE, particularly when conventional germline analysis is unrevealing.

## Materials and methods

Of 31 patients with cSLE under follow-up in March 2024, 26 provided written consent for genetic analysis and research use of clinical data and were enrolled; consent was unavailable for five. All fulfilled the 2012 Systemic Lupus International Collaborating Clinics classification criteria.

Whole-blood DNA underwent clinical hybrid-capture sequencing at the Kazusa DNA Research Institute on a NextSeq 2000 using P3 reagents (2 × 150-bp paired-end), following a published workflow (5). Reads were aligned to GRCh38 with Burrows-Wheeler Aligner 0.7.17, and germline variants were called with Genome Analysis Toolkit (GATK) 4.2.5.0. Minimum depth was 20×; target-region coverage was 100% in both patients. Neither low-VAF *KRAS* variant passed the GATK germline filter, but both were supported by additional callers and manual Integrative Genomics Viewer review before reporting. At Hiroshima University, BAM files generated by the Kazusa pipeline for all 26 patients were uniformly reassessed using a predefined research list of 576 genes based on the 2024 International Union of Immunological Societies classification, including *KRAS*. Secondary analysis used GATK 4.3.0.0, SAMtools and BCFtools 1.13, and snpEff and SnpSift 5.1d, with population frequency <0.01 and mappability >0.5. Variants were annotated against NM_004985.5. Because clinical panel composition changed across assay versions, uniform BAM reanalysis ensured consistent *KRAS* interrogation; residual capture-efficiency differences between versions cannot be excluded.

For both patients, initial and mosaicism-analysis samples were collected during clinically stable follow-up. DNA was extracted from blood with the QIAamp DNA Blood Mini Kit and from hair, nail, saliva, and urine with QIAamp DNA Investigator or Micro Kits (QIAGEN). At the Institute of Science Tokyo, *KRAS* c.40 and c.64 were amplified by first-round PCR using KOD FX Neo (TOYOBO) with primers 5′-ACA​CTC​TTT​CCC​TAC​ACG​ACG​CTC​TTC​CGA​TCT​AAA​GGT​ACT​GGT​GGA​GTA​TTT​GA-3′ and 5′-GTG​ACT​GGA​GTT​CAG​ACG​TGT​GCT​CTT​CCG​ATC​TGG​TCC​TGC​ACC​AGT​AAT​ATG​C-3′. Amplicons underwent second-round PCR, library preparation, and NextSeq 1000 sequencing at the Bioengineering Lab, using P1 600-cycle reagents (2 × 300-bp paired-end). FASTQ files were analyzed at the Institute of Science Tokyo. Reads were mapped to a GRCh38-derived *KRAS* reference with minimap2 v2.26-r1175 (-ax sr), sorted and indexed with SAMtools v1.19, and processed with BCFtools v1.19 mpileup using mapping-quality ≥20 and base-quality ≥30. VAF was calculated as alternate reads/(reference reads + alternate reads). Background was measured at c.64 in patient 2 and c.40 in patient 1 under identical filtering. Each specimen was analyzed once; dedicated negative controls and technical replicates were absent, and the analytical limit of detection was not established.

Variant nomenclature followed Human Genome Variation Society recommendations. ClinVar classifications were accessed on August 21, 2026, and CADD v1.6 provided supplementary in silico evidence. The Institute of Science Tokyo Institutional Review Board approved the study (G2000-103 and G2019-004); participants and parents or legal guardians provided written informed consent.

## Data Availability

Existing ClinVar records are NM_004985.5:c.64C>A, p.Gln22Lys (SCV007670263), and NM_004985.5:c.40G>A, p.Val14Ile (SCV007670264). Variant-level data are reported herein. Underlying sequencing data are not public because rare-disease genetic data may be identifiable. De-identified data may be available from the corresponding authors on reasonable requests, subject to institutional ethical approval and applicable data-sharing agreements.

## References

[1] Menzel, K., K. Novotna, N. Jeyakumar, C. Wolf, and M.A. Lee-Kirsch. 2024. Monogenic lupus – from gene to targeted therapy. Mol. Cell Pediatr. 11:8. 10.1186/s40348-024-00181-x PMC11393215 39264482

[2] Takagi, M., K. Shinoda, J. Piao, N. Mitsuiki, M. Takagi, K. Matsuda, H. Muramatsu, S. Doisaki, M. Nagasawa, T Morio, . 2011. Autoimmune lymphoproliferative syndrome-like disease with somatic KRAS mutation. Blood. 117:2887–2890. 10.1182/blood-2010-08-301515 21063026

[3] Law, S.M., S. Akizuki, A. Morinobu, and K. Ohmura. 2022. A case of refractory systemic lupus erythematosus with monocytosis exhibiting somatic KRAS mutation. Inflamm. Regen. 42:10. 10.1186/s41232-022-00195-w PMC8973904 35361277

[4] Loree, J.M., Y. Wang, M.A. Syed, A.V. Sorokin, O. Coker, J. Xiu, B.A. Weinberg, A.M. Vanderwalde, A. Tesfaye, V.M Raymond, . 2021. Clinical and functional characterization of atypical KRAS/NRAS mutations in metastatic colorectal cancer. Clin. Cancer Res. 27:4587–4598. 10.1158/1078-0432.CCR-21-0180 PMC8364867 34117033

[5] Fujiki, R., M. Ikeda, A. Yoshida, A. Maeda, Y. Yao, M. Nishimura, K. Matsushita, T. Ichikawa, T. Tanaka, H Morisaki, . 2018. Assessing the accuracy of variant detection in cost-effective gene panel testing by next-generation sequencing. J. Mol. Diagn. 20:572–582. 10.1016/j.jmoldx.2018.04.004 29953964

